# Association of PDCD6 polymorphisms with the risk of cancer: Evidence from a meta-analysis

**DOI:** 10.18632/oncotarget.25324

**Published:** 2018-05-15

**Authors:** Mohammad Hashemi, Gholamreza Bahari, Jarosław Markowski, Andrzej Małecki, Marek J. Łos, Saeid Ghavami

**Affiliations:** ^1^ Cellular and Molecular Research Center, Zahedan University of Medical Sciences, Zahedan, Iran; ^2^ Department of Clinical Biochemistry, School of Medicine, Zahedan University of Medical Sciences, Zahedan, Iran; ^3^ ENT Department, School of Medicine, Medical University of Silesia in Katowice, Katowice, Poland; ^4^ Faculty of Physiotherapy, The Jerzy Kukuczka Academy of Physical Education in Katowice, Katowice, Poland; ^5^ Department of Molecular Biology, School of Pharmacy with the Division of Laboratory Medicine in Sosnowiec, Medical University of Silesia in Katowice, Katowice, Poland; ^6^ Department of Human Anatomy and Cell Science, Max Rady College of Medicine, Rady Faculty of Health Sciences, University of Manitoba, Winnipeg, MB, Canada; ^7^ Health Policy Research Center, Institute of Health, Shiraz University of Medical Sciences, Shiraz, Iran; ^8^ Centre de Biophysique Moléculaire, CNRS, Rue Charles Sadron, Orleans, France

**Keywords:** PDCD6, meta-analysis, cancer, risk, endometrial cancer

## Abstract

This study was designed to evaluate the relationship between Programmed cell death protein 6 (PDCD6) polymorphisms and cancer susceptibility. The online databases were searched for relevant case-control studies published up to November 2017. Review Manage (RevMan) 5.3 was used to conduct the statistical analysis. The pooled odds ratio (OR) with its 95% confidence interval (CI) was employed to calculate the strength of association. Overall, our results indicate that PDCD6 rs3756712 T>G polymorphism was significantly associated with decreased risk of cancer under codominant (OR = 0.82, 95%CI = 0.70–0.96, *p* = 0.01, TG vs TT; OR = 0.53, 95%CI = 0.39-0.72, *p* < 0.0001, GG vs TT), dominant (OR = 0.76, 95%CI = 0.66-0.89, *p* = 0.0004, TG+GG vs TT), recessive (OR = 0.57, 95%CI = 0.43-0.78, *p* = 0.0003, GG vs TT+TG), and allele (OR = 0.76, 95%CI = 0.67–0.86, *p* < 0.00001, G vs T) genetic model. The finding did not support an association between rs4957014 T>G polymorphism of PDCD6, and different cancers risk.

## INTRODUCTION

Cancer is a major public health burden, with an estimate of 14.1 million new cancer cases and 8.2 million cancer-related deaths occurred globally in 2012 [3]. In 2018, 1,735,350 new cancer cases and 609,640 cancer deaths are projected to occur in the United States. It has been reported that over the past decade, the rate of incidence (2005–2014) was nearly linear and without any changes in women, and declined by approximately 2% annually in men, while the rate of cancer death (2006–2015) was declined by about 1.5% annually in both men and women [32]. It is estimated that in 2017, 1,688,780 new cancer cases was diagnosed and 600,920 cancer deaths are estimated to occurred in the United States [31]. Although significant progress has been reached in understanding the mechanism and pathogenesis of different types of cancers, the exact etiology is still not completely understood. Growing evidences indicate that cancer is a multifactorial disease caused by genetic background and environmental interactions [4, 8].

Apoptosis, also known as programmed cell death, is involved in physiological cell death [11]. Many factors contribute in the apoptotic pathway, including caspases, pro- and anti-apoptotic Bcl2 family members, and mitochondrial pro-apoptotic proteins [5, 6, 22]. Defects in the apoptosis machinery may lead to serious disease including cancer, autoimmune disease and drug resistance in tumors [5, 7, 26].

Programmed cell death protein 6 (*PDCD6*), located on chromosome 5p15.33 contains 43351 bp, is also known as apoptosis-linked gene-2 (*ALG-2*). *PDCD6* gene encodes a 22 kDa calcium-binding protein comprising five serially repetitive EF-hand structures. This protein is one of the prototypic members of the penta-EF-hand protein family. Initially, PDCD6 was considered as a pro-apoptotic protein contributing to T-cell receptor-, Fas-, and glucocorticoid- induced programmed cell death [30, 12], as well as endoplasmic reticulum stress induced apoptosis during organ formation [25, 18]. In recent years, some PDCD6-interacting proteins have been identified, including Peflin [14], Alix [21], Fas [12] and Annexin XI [28]. However none of them regulates PDCD6 activity, and such factor yet avaits to be identified. Alix and PDCD6 interaction with pro-caspase-8 potentiated cell death induction via tumor necrosis factor receptor 1 (TNFR1) [19]. Several studies have examined the expression of PDCD6 in clinical tumor tissues or cell lines, and found that PDCD6 has opposing effects in different tumors. PDCD6 expression was upregulated in tumor tissue samples from lung, breast, colon cancer, and ovarian cancer, which suggested that PDCD6 might be involved in maintenance of cellular viability [15, 10, 17, 33, 24]. In contrast, decreased PDCD6 expression was detected in non-small cell lung cancer (NSCLC), gastric cancer and HeLa cells [38, 40].

Recently, it has been shown that that miR-124-3p attenuated tumor metastasis by inhibiting PDCD6 expression, and that the miR-124-3p/PDCD6 signaling axis could potentially be a therapeutic target for patients with advanced breast cancer [43]. Another study showed that the over-expression of miR-124 suppressed PDCD6 expression, inhibited cell proliferation, migration and invasion, and induced apoptosis in SKOV3 and OCVAR3 cells *in vitro* [41]. Furthermorem it has been proposed that miR-183 may function as an oncogene that may increase childhood acute myeloid leukemia (AML) cell proliferation by targeting PDCD6 [37].

**Figure 1:**
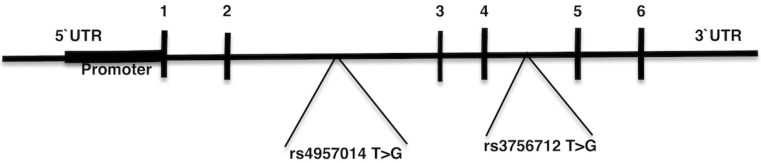
Map of the human *PDCD6* gene with polymorphisms positions indicated Exons 1–6 are numbered and represented by black boxes. Both of the rs3756712 T>G and rs4957014 T>G variants positioned in intron region.

Previous studies inspecting the association between *PDCD6* gene polymorphisms and cancer indicated inconclusive and contradictory results [9, 45, 44, 42]. Hence, we have performed a meta-analysis on all the published case-control studies to evaluate the association of *PDCD6* rs3756712 T>G and rs4957014 T>G gene polymorphisms with the risk of cancer. Maps of the human *PDCD6* gene with polymorphisms positions is illustrated in Figure 1.

## METHODOLOGY

**Figure 2:**
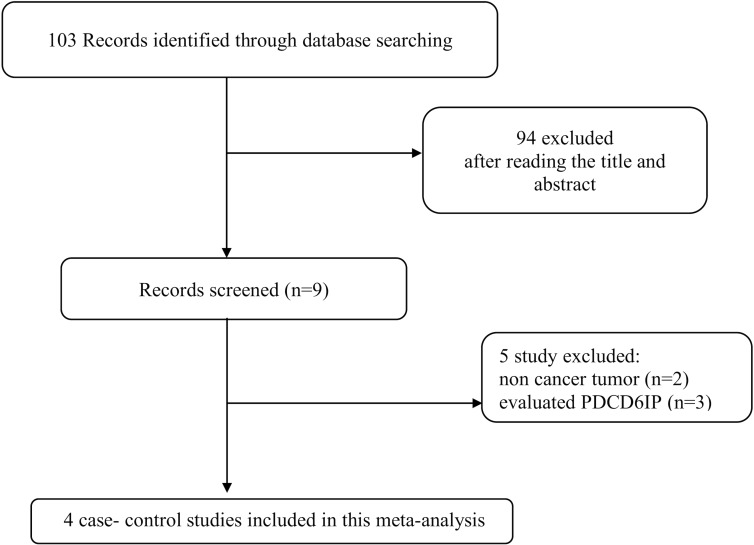
Flow chart of literature screening and selection in the meta-analysis

A comprehensive search in PubMed, Web of Science, Scopus, and Google Scholar databases was performed for all articles describing an association between *PDCD6* polymorphism and cancer risk published up to November 2017 without language restriction. The search strategy was “cancer, carcinoma, tumor, neoplasm”, “*PDCD6*, programmed cell death 6”, and “polymorphism, mutation, variant”. Figure 2 summarized the process of identifying eligible studies. Relevant studies, eligible for the meta-analysis must meet the following criteria: 1) Original case-control studies of the correlation between the *PDCD6* polymorphism and cancer; 2) studies provided sufficient information of the genotype frequencies of *PDCD6* polymorphism in both cases and controls; 3) the studies have not repeated reports in the same population. The criteria for exclusion were: 1) the articles that describe case reports, reviews, overlapped data, animal or mechanism studies for *PDCD6* polymorphism and cancer; 2) no genotype frequency or genotype information were provided for *PDCD6* polymorphism and cancer; 3) insufficient information for data extraction.

### Data extraction

Extraction of the data has been conducted by two independent scientists. The data were collected from each study including the first author’s name, publication year, ethnicity of participants, the sample size, and the genotype and allele frequencies of cases and controls.

### Statistical analysis

Meta-analysis was carried out using Revman 5.3 software, which was provided by the Cochrane Collaboration (Version 5.3. Copenhagen: The Nordic Cochrane Centre, the Cochrane Collaboration, 2014) and STATA 14.1 software (Stata Corporation, College Station, TX, USA). All of the data in the studies are dichotomous data, which has been expressed as odds ratios (ORs) with 95% confidence intervals (CIs) to assess the association between the polymorphisms and cancer. Hardy-Weinberg equilibrium (HWE) for each study was determined by the chi-square tests of control group data. Odds ratios (ORs) and 95% confidence intervals (CIs) were pooled to evaluate the association between the polymorphisms and risk of cancer. For each polymorphism the ORs were calculated for dominant, codominant, recessive, over-dominant, and allele genetic models. Heterogeneity also assessed using the I^2^ statistic, interpreted as the proportion of the total variation contributed by inter-study variation, and the Cochran chi-square *Q*-test, with a significance level of *P* < 0.10 and *I*^2^ > 50%. When significant heterogeneity values were returned, the random-effects model (the DerSimonian and Laird method) was used to estimate pooled ORs. Otherwise, the fixed-effects model (the Mantel-Haenszel method) was employed. The significance of the pooled OR was assessed by the *Z*-test, and *P* < 0.05 was considered to be statistically significant.

Publication bias was evaluated by funnel plot. The degree of asymmetry was measured using Egger’s linear regression test; *p* < 0.05 was considered significant publication bias [2]. The characteristics and relevant data of the included studies are shown in Table 1. The genotypes of *PDCD6* polymorphisms in controls were all in accordance with HWE (*P* > 0.05) except for Zhou *et al.* [45] (Table 1).

**Table 1:** Distribution information of genotypes and alleles of all studies included in the meta-analysis for rs3756712 T>G and rs4957014 T>G polymorphisms of programmed cell death 6 (PDCD6)

Author	Year	Country	Ethnicity	Cancer type	Source of control	Genotyping method	Case/control	cases					Controls					HWE
rs3756712								TT	GT	GG	T	G	TT	GT	GG	T	G	
Yuan	2017	China	Asian	Endometrial Cancer	HB	PCR-RFLP	238/518	153	70	15	376	100	290	184	44	764	272	0.060
Zhou	2015	China	Asian	Cervical squamous cell carcinoma	HB	PCR-RFLP	328/541	202	109	17	513	143	298	195	48	791	291	0.053
Zhou	2014	China	Asian	Bladder cancer	HB	PCR-RFLP	332/509	214	101	17	529	135	279	183	47	741	277	0.037
He	2012	China	Asian	Lung cancer	HB	PCR-RFLP	302/306	168	120	14	456	148	168	111	27	447	165	0.167
rs4957014								TT	GT	GG	T	G	TT	GT	GG	T	G	
Yuan	2017	China	Asian	Endometrial Cancer	HB	PCR-RFLP	238/518	83	131	24	297	179	234	231	53	699	337	0.720
Zhou	2015	China	Asian	Cervical squamous cell carcinoma	HB	PCR-RFLP	328/541	130	142	56	402	254	243	246	52	732	350	0.365
Zhou	2014	China	Asian	Bladder cancer	HB	PCR-RFLP	332/509	170	125	37	465	199	229	232	48	690	328	0.325
He	2012	China	Asian	Lung cancer	HB	PCR-RFLP	302/306	155	124	23	434	170	134	136	36	404	208	0.868

HB, hospital based.

## RESULTS

### Association of PDCD6 rs3756712 T>G and rs4957014 T>G polymorphisms and cancer risk

**Figure 3:**
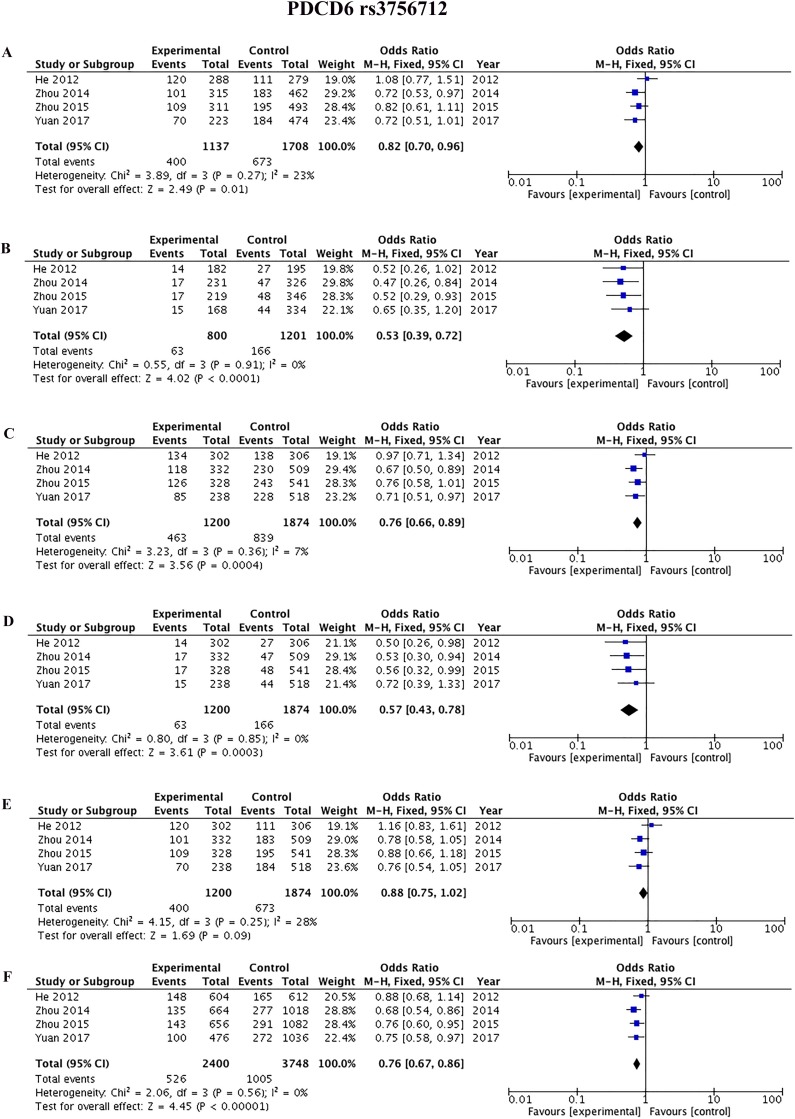
Forest plots of the association between cancer risk and the rs3756712 T>G polymorphism in the overall study population under the following models (**A**) TG *vs* TT, (**B**) GG *vs* TT, (**C**) TG+GG *vs* TT, (**D**) GG *vs* TT+TG, (**E**) TG *vs* TT+GG, and (**F**) G *vs* T.

**Figure 4:**
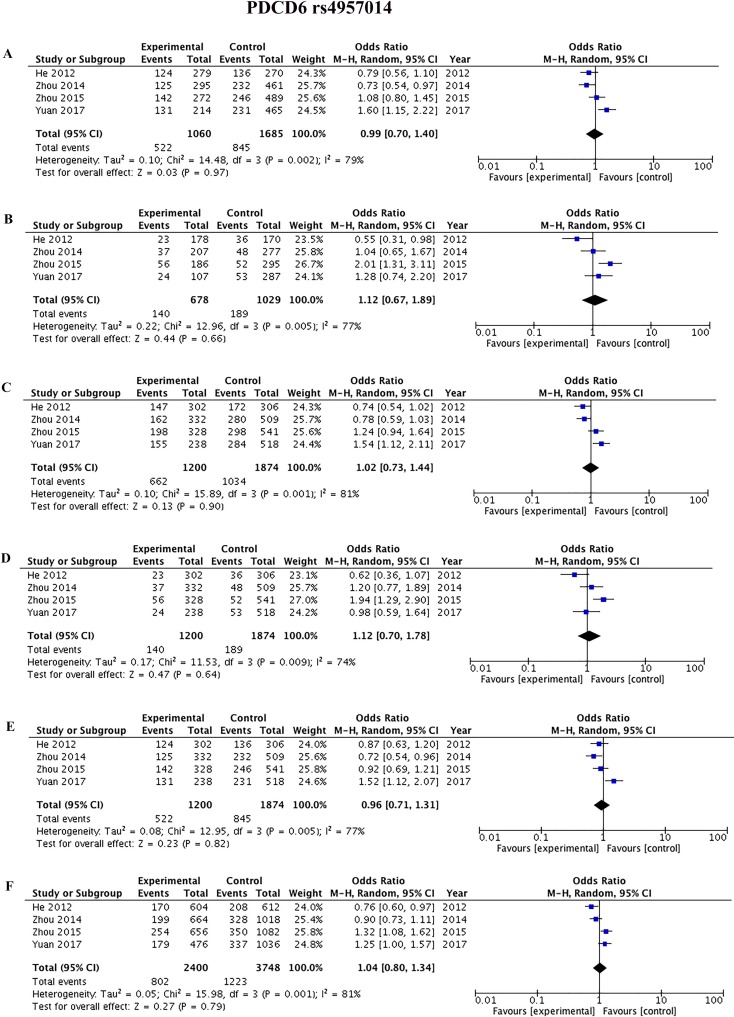
Forest plots of the association between cancer risk and the rs4957014 T>G polymorphism in the overall study population under the following models (**A**) TG *vs* TT, (**B**) GG *vs* TT, (**C**) TG+GG *vs* TT, (**D**) GG *vs*TT+TG, (**E**) TG *vs* TT+GG, and (**F**) G *vs* T.

Four studies [42, 45, 44, 9] reported the association between rs3756712 and rs4957014 polymorphisms and cancer. As shown in Figure 3 and Table 2, the results showed that *PDCD6* rs3756712 T>G polymorphism was significantly associated with decreased risk of cancer under codominant (OR = 0.82, 95%CI = 0.70–0.96, *p* = 0.01, TG vs TT; OR = 0.53, 95%CI = 0.39–0.72, *p* < 0.0001, GG vs TT), dominant (OR = 0.76, 95%CI = 0.66–0.89, *p* = 0.0004, TG+GG vs TT), recessive (OR = 0.57, 95%CI = 0.43–0.78, *p* = 0.0003, GG vs TT+TG), and allele (OR = 0.76, 95%CI = 0.67–0.86, *p*<0.00001, G vs T) genetic model. Regarding rs4957014 T>G polymorphism of *PDCD6*, the finding did not support an association between rs4957014 T>G polymorphism and cancer risk (Figure 4 and Table 2).

**Table 2:** Meta-analysis of the association between PDCD6 rs3756712 T>G and rs4957014 T>G polymorphisms and cancer risk

SNP	Genetic model	OR	95%CI	*p*	Heterogenecity I^2^ (%)	Egger’s test *P*	Begg’s test *P*
rs3756712							
	TG vs TT	0.82	0.70–0.96	0.01	23	0.656	1.00
	GG vs TT	0.53	0.39–0.72	<0.0001	0	0.759	1.00
	TG+GG vs TT	0.76	0.66–0.89	0.0004	7	0.477	0.497
	GG vs TT+TG	0.57	0.43–0.78	0.0003	0	0.877	0.497
	TG vs TT+GG	0.88	0.75–1.02	0.09	28	0.729	0.497
	G vs T	0.76	0.67–0.86	<0.00001	0	0.448	1.00
rs4957014							
	TG vs TT	0.99	0.70–1.40	0.97	79	0.661	0.497
	GG vs TT	1.12	0.67–1.89	0.66	77	0.190	0.174
	TG+GG vs TT	1.02	0.73–1.44	0.90	81	0.865	0.497
	GG vs TT+TG	1.12	0.70–1.78	0.64	74	0.018	0.042
	TG vs TT+GG	0.96	0.71–1.31	0.82	77	0.570	1.00
	G vs T	1.04	0.80–1.34	0.79	81	0.373	0.174

### Heterogeneity and publication bias

**Figure 5:**
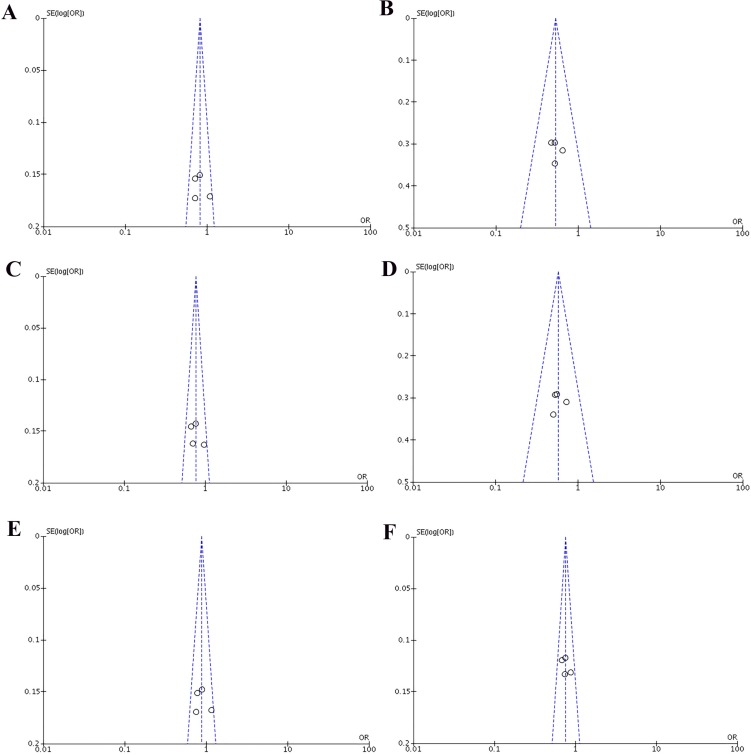
Funnel plots of the association between cancer risk and the rs3756712 T>G polymorphism in the overall study population under the following models (**A**) TG *vs* TT, (**B**) GG *vs* TT, (**C**) TG+GG *vs* TT, (**D**) GG *vs* TT+TG, (**E**) TG *vs* TT+GG, and (**F**) G *vs* T.

**Figure 6:**
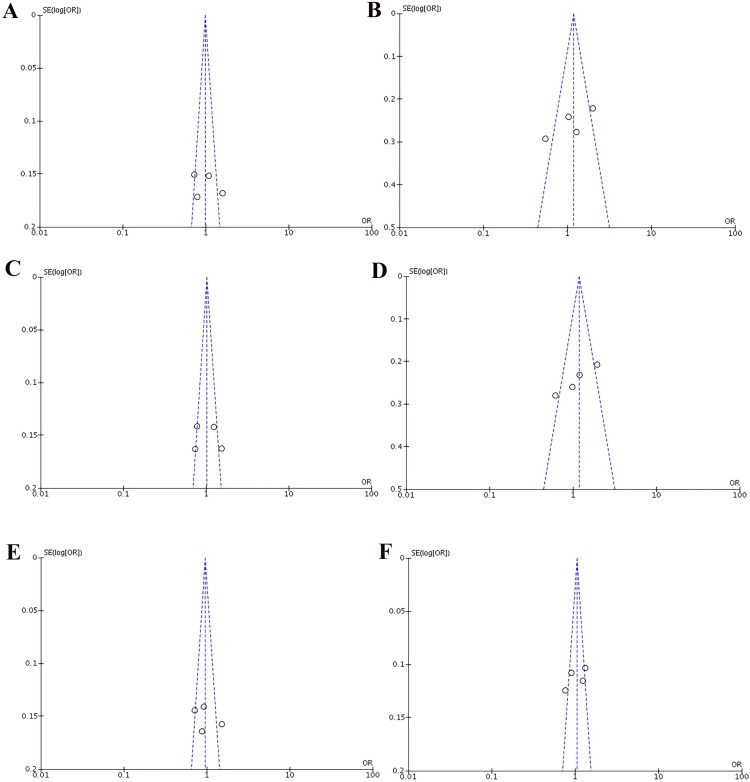
Funnel plots of the association between cancer risk and the rs4957014 T>G polymorphism in the overall study population under the following models (**A**) TG *vs* TT, (**B**) GG *vs* TT, (**C**) TG+GG *vs* TT, (**D**) GG *vs* TT+TG, (**E**) TG *vs* TT+GG, and (**F**) G *vs* T.

Heterogeneity of the included studies concerning each polymorphism is shown in Table 2. A funnel plot was generated as a visual aid to detect risk of publication bias (Figures 5 and 6). Regarding rs3756712 variant, Egger’s linear regression analysis suggested no publication bias for this meta-analysis of the codominant, dominant, recessive, overdominanat and allele model (all *P*-values for bias > 0.05). For rs4957014 polymorphism, the findings indicated that the publication bias exist only for recessive model (Table 2).

### Sensitivity analysis

**Figure 7:**
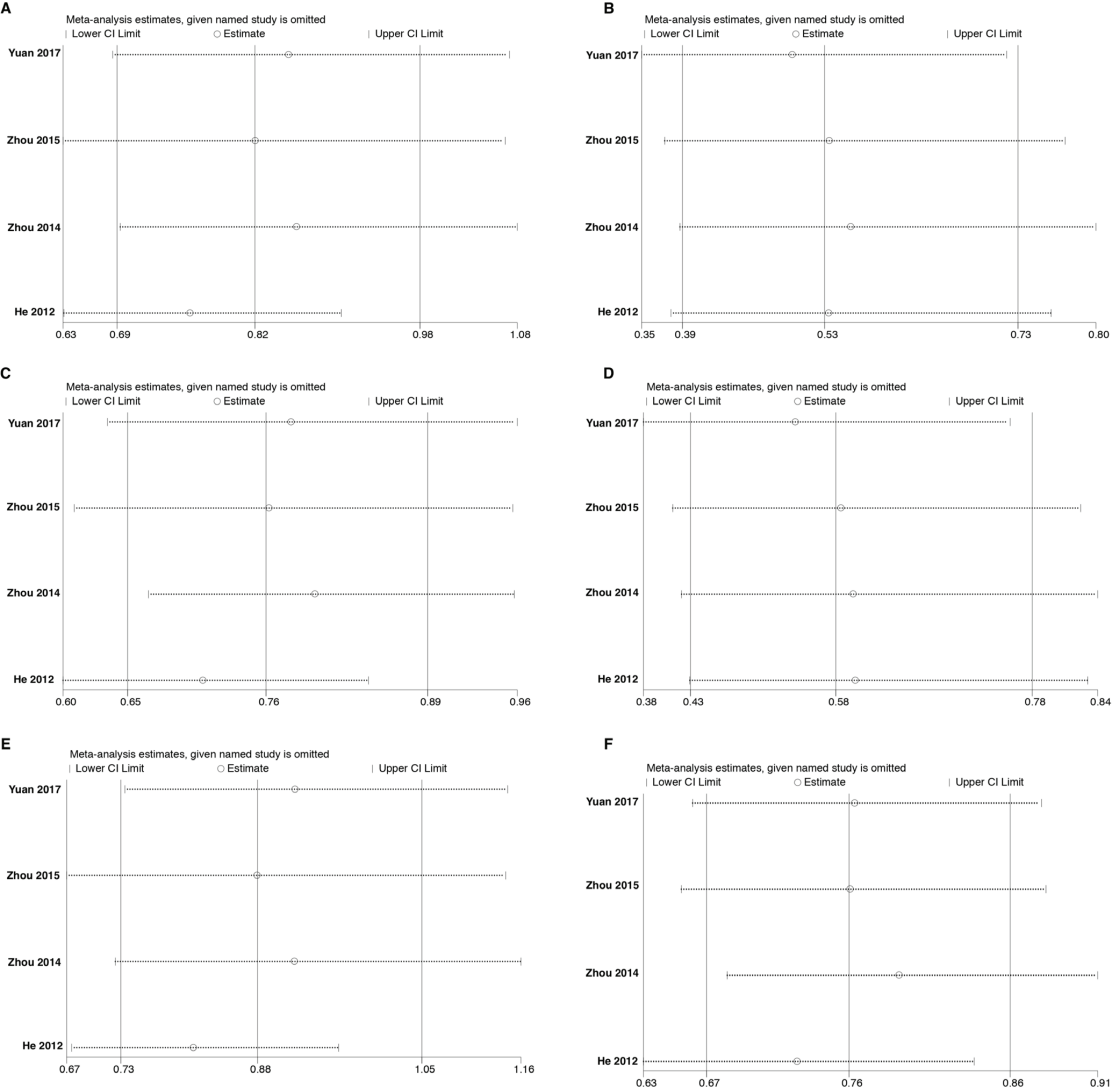
Sensitivity analyses for studies on *PDCD6* rs3756712 T>G using different genetic models (**A**) TG vs TT, (**B**) GG vs TT, (**C**) TG+GG vs TT, (**D**) GG vs TT+TG, (**E**) TG vs TT+GG, and (**F**) G vs T.

**Figure 8:**
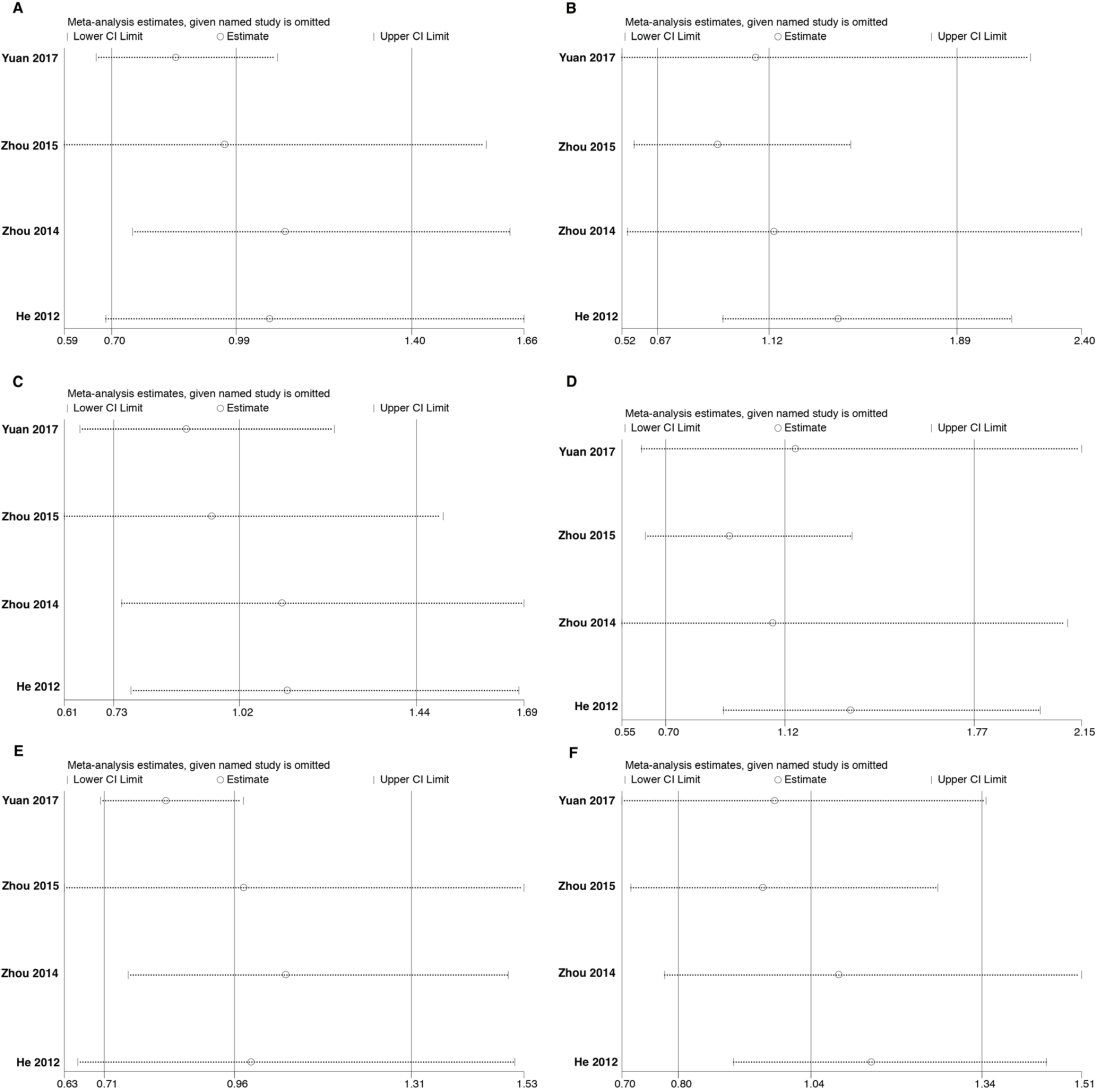
Sensitivity analyses for studies on *PDCD6* rs4957014 T>G using different genetic models (**A**) TG vs TT, (**B**) GG vs TT, (**C**) TG+GG vs TT, (**D**) GG vs TT+TG, (**E**) TG vs TT+GG, and (**F**) G vs T.

Sensitivity analysis was done using the method of eliminating studies one by one to verify whether our results were influenced by each included study. For rs3756712 variant, the pooled ORs were not considerably altered except in codominant heterozygous model (TG vs TT) and overdominant (TG vs TT+GG) model (Figure 7). Regarding rs4957014 variant, the pooled ORs altered only in overdominant model (Figure 8). Therefore, the results confirmed that the present meta-analysis was relatively stable and reliable.

## DISCUSSION

PDCD6, a proapoptotic protein, is a Ca^2+^ binding protein of the EF-hand type belonging to the subfamily of penta-EF-hand (PEF) protein that is required for the induction of apoptosis by a variety of stimuli [35, 20]. Binding of Ca^2+^ to PDCD6 induces a conformational change [34], which enables the interaction with several proteins, including ALG-2-interacting protein X (ALIX) [36], Sec31A [39, 16], and annexin A11 [28, 29]. Several lines of evidence suggest that interacting partner of PDCD6 is ALIX/AIP1, an adaptor protein, which has been implicated in apoptotic signaling [23, 13].

The pathogenesis of carcinogenesis involves environmental factors, molecular signaling pathways, and host genetic factors [1, 6]. Since single nucleotide polymorphism (SNP) is the main cause of human genetic variation, the connection between SNPs and individual risk of cancer has drawn considerable attention [27, 8]. As we know, limited polymorphisms have been reported about *PDCD6* gene. Several investigations have been done to find the possible association between two tag SNPs of *PDCD6*, rs3756712 and rs4957014 polymorphisms, and various cancer risk [45, 9, 44, 42]. Zhou *et al.* [45] reported that rs3756712 and rs4957014 polymorphisms of *PDCD6* significantly decreased the risk of bladder cancer. He *et al.* [9] have found no significant association between *PDCD6* rs3756712 variant and risk of Non-small cell lung cancer (NSCLC). Their findings proposed that rs4957014 variant of *PDCD6* significantly decreased the risk of NSCLC. On the other hand, it has been revealed that rs3756712 and rs4957014 polymorphisms of *PDCD6* significantly increased the risk of cervical squamous cell carcinoma (CSCC) [44]. Recently, Yuan *et al.* [42] investigated the association between rs3756712 and rs4957014 polymorphisms of *PDCD6* and risk of CSCC. Their findings suggested that both of the variants significantly increased the risk of CSCC.

Due to contrasting findings about *PDCD6* polymorphism in certain types of cancers, the present meta-analysis was performed to evaluate the impact of two SNPs of *PDCD6* on cancer risk. To our knowledge, this is the first meta-analysis evaluating the impact of *PDCD6* polymorphisms on cancer. The attained results suggest that the *PDCD6* rs3756712 T>G polymorphism significantly decreased the risk of cancer under codominant, dominant, recessive, and allele genetic model. The findings did not support an association between rs4957014 T>G polymorphism of *PDCD6* and cancer risk.

In conclusion, to our knowledge, this fist the present study suggested that there is an association between the *PDCD6* rs3756712 T>G polymorphism and cancer. The rs3756712 T>G variant may be a potential marker for cancer. Further well-designed case-control studies with a larger sample size and different ethnicities should be done to confirm the findings.

## Abbreviations

ALG-2: apoptosis-linked gene-2
AIP1: ALG-2-interacting protein 1
ALIX: ALG-2-interacting protein X
CSCC: cervical squamous cell carcinoma
CI: confidence interval
HWE: Hardy-Weinberg equilibrium
NSCLC: non-small cell lung cancer
OR: odds ratio
PEF: penta-EF-hand
PDCD6: Programmed cell death protein 6
RevMan: Review Manager
SNP: since single nucleotide polymorphism

## References

[1] Alizadeh J, Zeki AA, Mirzaei N, Tewary S, Rezaei Moghadam A, Glogowska A, Nagakannan P, Eftekharpour E, Wiechec E, Gordon JW, Xu FY, Field JT, Yoneda KY (2017). Mevalonate Cascade Inhibition by Simvastatin Induces the Intrinsic Apoptosis Pathway via Depletion of Isoprenoids in Tumor Cells. Sci Rep.

[2] Egger M, Davey Smith G, Schneider M, Minder C (1997). Bias in meta-analysis detected by a simple, graphical test. BMJ.

[3] Ferlay J, Soerjomataram I, Dikshit R, Eser S, Mathers C, Rebelo M, Parkin DM, Forman D, Bray F (2015). Cancer incidence and mortality worldwide: sources, methods and major patterns in GLOBOCAN 2012. Int J Cancer.

[4] Foulkes WD (2008). Inherited susceptibility to common cancers. N Engl J Med.

[5] Ghavami S, Hashemi M, Ande SR, Yeganeh B, Xiao W, Eshraghi M, Bus CJ, Kadkhoda K, Wiechec E, Halayko AJ, Los M (2009). Apoptosis and cancer: mutations within caspase genes. J Med Genet.

[6] Ghavami S, Shojaei S, Yeganeh B, Ande SR, Jangamreddy JR, Mehrpour M, Christoffersson J, Chaabane W, Moghadam AR, Kashani HH, Hashemi M, Owji AA, Los MJ (2014). Autophagy and apoptosis dysfunction in neurodegenerative disorders. Prog Neurobiol.

[7] Hashemi M, Kroczak TJ (2005). Apoptosis and autoimmune disease. Curr Med Chem Anti Inflamm Anti Allergy Agents.

[8] Hassanzarei S, Hashemi M, Sattarifard H, Hashemi SM, Bahari G, Ghavami S (2017). Genetic polymorphisms of HOTAIR gene are associated with the risk of breast cancer in a sample of southeast Iranian population. Tumour Biol.

[9] He YQ, Zhou B, Shi SQ, Zhang L, Li WM (2012). Genetic variation in PDCD6 and susceptibility to lung cancer. Asian Pac J Cancer Prev.

[10] Hoj BR, la Cour JM, Mollerup J, Berchtold MW (2009). ALG-2 knockdown in HeLa cells results in G2/M cell cycle phase accumulation and cell death. Biochem Biophys Res Commun.

[11] Hombach-Klonisch S, Mehrpour M, Shojaei S, Harlos C, Pitz M, Hamai A, Siemianowicz K, Likus W, Wiechec E, Toyota BD, Hoshyar R, Seyfoori A, Sepehri Z (2018). Glioblastoma and chemoresistance to alkylating agents: Involvement of apoptosis, autophagy, and unfolded protein response. Pharmacol Ther.

[12] Jung YS, Kim KS, Kim KD, Lim JS, Kim JW, Kim E (2001). Apoptosis-linked gene 2 binds to the death domain of Fas and dissociates from Fas during Fas-mediated apoptosis in Jurkat cells. Biochem Biophys Res Commun.

[13] Kaul Z, Chakrabarti O (2017). Tumor susceptibility gene 101 regulates predisposition to apoptosis via ESCRT machinery accessory proteins. Mol Biol Cell.

[14] Kitaura Y, Matsumoto S, Satoh H, Hitomi K, Maki M (2001). Peflin and ALG-2, members of the penta-EF-hand protein family, form a heterodimer that dissociates in a Ca2+-dependent manner. J Biol Chem.

[15] la Cour JM, Hoj BR, Mollerup J, Simon R, Sauter G, Berchtold MW (2008). The apoptosis linked gene ALG-2 is dysregulated in tumors of various origin and contributes to cancer cell viability. Mol Oncol.

[16] la Cour JM, Mollerup J, Berchtold MW (2007). ALG-2 oscillates in subcellular localization, unitemporally with calcium oscillations. Biochem Biophys Res Commun.

[17] la Cour JM, Mollerup J, Winding P, Tarabykina S, Sehested M, Berchtold MW (2003). Up-regulation of ALG-2 in hepatomas and lung cancer tissue. Am J Pathol.

[18] Mahul-Mellier AL, Hemming FJ, Blot B, Fraboulet S, Sadoul R (2006). Alix, making a link between apoptosis-linked gene-2, the endosomal sorting complexes required for transport, and neuronal death *in vivo*. J Neurosci.

[19] Mahul-Mellier AL, Strappazzon F, Petiot A, Chatellard-Causse C, Torch S, Blot B, Freeman K, Kuhn L, Garin J, Verna JM, Fraboulet S, Sadoul R (2008). Alix and ALG-2 are involved in tumor necrosis factor receptor 1-induced cell death. J Biol Chem.

[20] Maki M, Narayana SV, Hitomi K (1997). A growing family of the Ca2+-binding proteins with five EF-hand motifs. Biochem J.

[21] Missotten M, Nichols A, Rieger K, Sadoul R (1999). Alix, a novel mouse protein undergoing calcium-dependent interaction with the apoptosis-linked-gene 2 (ALG-2) protein. Cell Death Differ.

[22] Mokarram P, Albokashy M, Zarghooni M, Moosavi MA, Sepehri Z, Chen QM, Hudecki A, Sargazi A, Alizadeh J, Moghadam AR, Hashemi M, Movassagh H, Klonisch T (2017). New frontiers in the treatment of colorectal cancer: Autophagy and the unfolded protein response as promising targets. Autophagy.

[23] Odorizzi G (2006). The multiple personalities of Alix. J Cell Sci.

[24] Qin J, Li D, Zhou Y, Xie S, Du X, Hao Z, Liu R, Liu X, Liu M, Zhou J (2017). Apoptosis-linked gene 2 promotes breast cancer growth and metastasis by regulating the cytoskeleton. Oncotarget.

[25] Rao RV, Poksay KS, Castro-Obregon S, Schilling B, Row RH, del Rio G, Gibson BW, Ellerby HM, Bredesen DE (2004). Molecular components of a cell death pathway activated by endoplasmic reticulum stress. J Biol Chem.

[26] Rashedi I, Panigrahi S, Ezzati P, Ghavami S, Los M (2007). Autoimmunity and apoptosis—therapeutic implications. Curr Med Chem.

[27] Risch N, Merikangas K (1996). The future of genetic studies of complex human diseases. Science.

[28] Satoh H, Shibata H, Nakano Y, Kitaura Y, Maki M (2002). ALG-2 interacts with the amino-terminal domain of annexin XI in a Ca(2+)-dependent manner. Biochem Biophys Res Commun.

[29] Shibata H, Kanadome T, Sugiura H, Yokoyama T, Yamamuro M, Moss SE, Maki M (2015). A new role for annexin A11 in the early secretory pathway via stabilizing Sec31A protein at the endoplasmic reticulum exit sites (ERES). J Biol Chem.

[30] Shibata H, Yamada K, Mizuno T, Yorikawa C, Takahashi H, Satoh H, Kitaura Y, Maki M (2004). The penta-EF-hand protein ALG-2 interacts with a region containing PxY repeats in Alix/AIP1, which is required for the subcellular punctate distribution of the amino-terminal truncation form of Alix/AIP1. J Biochem.

[31] Siegel RL, Miller KD, Jemal A (2017). Cancer Statistics, 2017. CA Cancer J Clin.

[32] Siegel RL, Miller KD, Jemal A (2018). Cancer statistics, 2018. CA Cancer J Clin.

[33] Su D, Xu H, Feng J, Gao Y, Gu L, Ying L, Katsaros D, Yu H, Xu S, Qi M (2012). PDCD6 is an independent predictor of progression free survival in epithelial ovarian cancer. J Transl Med.

[34] Suzuki H, Kawasaki M, Inuzuka T, Okumura M, Kakiuchi T, Shibata H, Wakatsuki S, Maki M (2008). Structural basis for Ca2+ -dependent formation of ALG-2/Alix peptide complex: Ca2+/EF3-driven arginine switch mechanism. Structure.

[35] Vito P, Lacana E, D'Adamio L (1996). Interfering with apoptosis: Ca(2+)-binding protein ALG-2 and Alzheimer's disease gene ALG-3. Science.

[36] Vito P, Pellegrini L, Guiet C, D'Adamio L (1999). Cloning of AIP1, a novel protein that associates with the apoptosis-linked gene ALG-2 in a Ca2+-dependent reaction. J Biol Chem.

[37] Wang X, Zuo D, Yuan Y, Yang X, Hong Z, Zhang R (2017). MicroRNA-183 promotes cell proliferation via regulating programmed cell death 6 in pediatric acute myeloid leukemia. J Cancer Res Clin Oncol.

[38] Yamada Y, Arao T, Gotoda T, Taniguchi H, Oda I, Shirao K, Shimada Y, Hamaguchi T, Kato K, Hamano T, Koizumi F, Tamura T, Saito D (2008). Identification of prognostic biomarkers in gastric cancer using endoscopic biopsy samples. Cancer Sci.

[39] Yamasaki A, Tani K, Yamamoto A, Kitamura N, Komada M (2006). The Ca2+-binding protein ALG-2 is recruited to endoplasmic reticulum exit sites by Sec31A and stabilizes the localization of Sec31A. Mol Biol Cell.

[40] Yoon JH, Choi YJ, Kim SG, Nam SW, Lee JY, Park WS (2012). Programmed cell death 6 (PDCD6) as a prognostic marker for gastric cancers. Tumour Biol.

[41] Yuan L, Li S, Zhou Q, Wang D, Zou D, Shu J, Huang Y (2017). MiR-124 inhibits invasion and induces apoptosis of ovarian cancer cells by targeting programmed cell death 6. Oncol Lett.

[42] Yuan M, Song Y, You D, Li Q, Zhang Y, Zhou B, Zhang L, Xi M (2017). Association between single nucleotide polymorphisms in the programmed cell death 6 gene and the risk of endometrial cancer in Chinese Han women. Int J Clin Exp Pathol.

[43] Zhang L, Chen X, Liu B, Han J (2018). MicroRNA-124-3p directly targets PDCD6 to inhibit metastasis in breast cancer. Oncol Lett.

[44] Zhou B, Bai P, Xue H, Zhang Z, Shi S, Zhang K, Wang Y, Wang K, Quan Y, Song Y, Zhang L (2015). Single nucleotide polymorphisms in PDCD6 gene are associated with the development of cervical squamous cell carcinoma. Fam Cancer.

[45] Zhou B, Zhang P, Tang T, Zhang K, Wang Y, Song Y, Liao H, Zhang L (2014). Prognostic value of PDCD6 polymorphisms and the susceptibility to bladder cancer. Tumour Biol.

