# Correction: Association of PDCD6 polymorphisms with the risk of cancer: Evidence from a meta-analysis

**DOI:** 10.18632/oncotarget.27483

**Published:** 2020-03-03

**Authors:** Mohammad Hashemi, Gholamreza Bahari, Jarosław Markowski, Andrzej Małecki, Marek J. Łos, Saeid Ghavami

**Affiliations:** ^1^ Cellular and Molecular Research Center, Zahedan University of Medical Sciences, Zahedan, Iran; ^2^ Department of Clinical Biochemistry, School of Medicine, Zahedan University of Medical Sciences, Zahedan, Iran; ^3^ ENT Department, School of Medicine, Medical University of Silesia in Katowice, Katowice, Poland; ^4^ Faculty of Physiotherapy, The Jerzy Kukuczka Academy of Physical Education in Katowice, Katowice, Poland; ^5^ Department of Molecular Biology, School of Pharmacy with the Division of Laboratory Medicine in Sosnowiec, Medical University of Silesia in Katowice, Katowice, Poland; ^6^ Department of Human Anatomy and Cell Science, Max Rady College of Medicine, Rady Faculty of Health Sciences, University of Manitoba, Winnipeg, MB, Canada; ^7^ Health Policy Research Center, Institute of Health, Shiraz University of Medical Sciences, Shiraz, Iran; ^8^ Centre de Biophysique Moléculaire, CNRS, Rue Charles Sadron, Orleans, France

**This article has been corrected:** The Acknowledgements section has been updated. The proper text is as follows:

**ACKNOWLEDGMENTS**

SG was supported by CHRIM operating grant, Health Science Foundation general operating grant and Research Manitoba New Investigator operating grant. MJŁ kindly acknowledges the support from LE STUDIUM Institute for Advanced Studies (region Centre-Val de Loire, France) through its Smart Loire Valley General Program, cofunded by the Marie Sklodowska-Curie Actions, grant #665790.

Original article: Oncotarget. 2018; 9:24857–24868. 24857-24868. https://doi.org/10.18632/oncotarget.25324

